# Ribonucleotide reductase and thymidylate synthase or exogenous deoxyribonucleosides reduce DNA damage and senescence caused by C-MYC depletion

**DOI:** 10.18632/aging.100512

**Published:** 2012-12-13

**Authors:** Sudha Mannava, Kalyana C. Moparthy, Linda J. Wheeler, Katerina I. Leonova, Joseph A. Wawrzyniak, Anna Bianchi-Smiraglia, Albert E. Berman, Sheryl Flanagan, Donna S. Shewach, Nathalie C. Zeitouni, Andrei V. Gudkov, Christopher K. Mathews, Mikhail A. Nikiforov

**Affiliations:** ^1^ Department of Cell Stress Biology, Roswell Park Cancer Institute, Buffalo, NY 14263, USA; ^2^ Department of Dermatology, Roswell Park Cancer Institute, Buffalo, NY 14263, USA; ^3^ Department of Biochemistry and Biophysics, Oregon State University, Corvallis, OR 97331, USA; ^4^ V.N. Orekhovich Institute of Biomedical Chemistry RAMS, 119121 Moscow, Russia; ^5^ Department of Pharmacology, University of Michigan, Ann Arbor, MI 48109, USA

**Keywords:** myc, oncogene-induced senescence, melanoma, thymidylate synthase, ribonucleotide reductase, dNTP

## Abstract

The down-regulation of dominant oncogenes, including C-MYC, in tumor cells often leads to the induction of senescence via mechanisms that are not completely identified. In the current study, we demonstrate that MYC-depleted melanoma cells undergo extensive DNA damage that is caused by the underexpression of thymidylate synthase (TS) and ribonucleotide reductase (RR) and subsequent depletion of deoxyribonucleoside triphosphate pools. Simultaneous genetic inhibition of TS and RR in melanoma cells induced DNA damage and senescence phenotypes very similar to the ones caused by MYC-depletion. Reciprocally, overexpression of TS and RR in melanoma cells or addition of deoxyribo-nucleosides to culture media substantially inhibited DNA damage and senescence-associated phenotypes caused by C-MYC depletion. Our data demonstrate the essential role of TS and RR in C-MYC-dependent suppression of senescence in melanoma cells.

## INTRODUCTION

In normal cells a form of irreversible proliferation arrest, termed senescence, is induced in response to aberrant activation of oncogenes and therefore can be considered an intrinsic mechanism for suppressing tumor progression [1-3]. In some types of tumor cells, a similar form of proliferation arrest can be caused by tumor suppressors or DNA damaging agents [4, 5]. Recently, we have demonstrated that depletion of C-MYC in human melanoma cells led to the induction of senescence-associated phenotypes that were very similar to that of normal melanocytes undergoing oncogene-induced senescence (OIS) [6]. These phenotypes included permanent growth arrest, elevated activity of senescence-associated β-galactosidase (SA-β-Gal), and changes in histone modifications [6]. Moreover, like in normal melanocytes, senescence phenotypes in MYC-depleted melanoma cells depended on constitutively active BRAF or NRAS oncoproteins and did not depend on the status of p53 or p16^INK4A^[6].

C-MYC is a transcription factor that regulates the expression of more than 3,000 genes involved in a large variety of cellular processes including the cell cycle progression, metabolism and apoptosis [7-9]. Previously, we have identified three *bona fide* MYC target genes that were down-regulated shortly after shRNA-mediated depletion of C-MYC: thymidylate synthase (TS), inosine monophosphate dehydrogenase 2 (IMPDH2), and phosphoribosyl pyrophosphate synthetase 2 (PRPS2)[10]. Ectopic co-expression of TS, IMPDH2, and PRPS2 cDNAs in MYC-depleted cells provided only a partial and short-term delay in proliferation arrest [10], indicating that still unidentified C-MYC target genes were required for MYC-dependent suppression of senescence in melanoma cells.

In a separate study, we demonstrated that ectopic co-expression of TS and ribonucleotide reductase (RR), another rate-limiting enzyme for the de novo deoxyribonucleotide biosynthesis [11], suppressed oncogene-induced senescence in normal human fibroblasts [12]. Here, we investigate the role of TS and RR in C-MYC-dependent phenotypes in human melanoma cells and demonstrate that both enzymes are required for the mediation of C-MYC-dependent suppression of senescence in these cells.

## RESULTS

### MYC-depleted senescent melanoma cells under-express TS and RR

**Figure 1:**
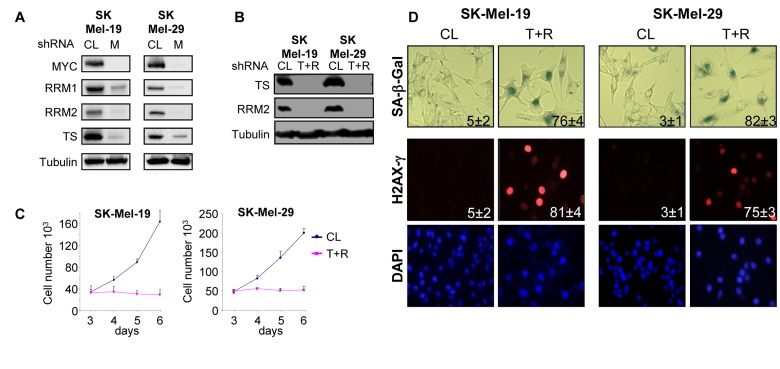
Deficiency of TS and RRM2 induces senescence in melanoma cells (**A**) Cells were infected with control (CL) or MYC shRNA (M) lentiviral vectors. Five days post-infection, cells were harvested, lysed and total protein extracts were probed in western blotting with the antibodies indicated on the left. (**B**) Cells were infected with control shRNA lentiviral vector (V) or with a combination of vectors carrying shRNAs for TS or RRM2 shRNAs (T+R). Five days post-infection, cells were harvested, lysed and total protein extracts were probed in western blotting with the antibodies indicated on the left. (**C**) Cells were infected as in (**A**). Two days after infection cells were plated in 12-well plate in duplicate and counted every day for 3 days. Numbers below the graph correspond to the days post-infection. (**D**) Cells were infected as in (**B**). Five days post-infection, cells were fixed and processed to visualize SA-β-Gal activity, or stained with H2AX-γ-specific antibodies and DAPI. Numbers indicate the percentage of SA-β-Gal-positive cells in each population. The numbers were derived from counting cells in several view fields.

We started with the analysis of the expression pattern of TS, RRM1 and RRM2 (two subunits of RR) in SK-Mel-19 and SK-Mel-29 cells 6 days post-infection with control or MYC shRNAs, i.e. at the time point when the majority of MYC-depleted cells underwent senescence [6]. As shown in Figure 1A, levels of all three proteins were significantly reduced in MYC-depleted cells. Next, to determine whether the depletion of these enzymes exerts effects similar to C-MYC depletion, SK-Mel-19 and SK-Mel-29 cells were transduced with control shRNA lentiviral vector or co-transduced with vectors carrying shRNAs for TS and RRM2 (Figure 1B).

The depletion of both TS and RRM2 induced proliferation arrest in the majority of cells (Figure 1C), although a small subset of cells underwent apoptosis. Melanoma cells depleted of TS and RRM2 demonstrated substantial DNA damage (detected by antibodies to phosphorylated histone H2AX [H2AX-γ]) and increased activity of SA-β-Gal (Figure 1D). Therefore, concomitant inhibition of TS and RR induced senescence-associated phenotypes that were similar to those detected in MYC-depleted cells [6].

### Overexpression of TS and RR suppresses senescence phenotypes in MYC-depleted melanoma cells

**Figure 2:**
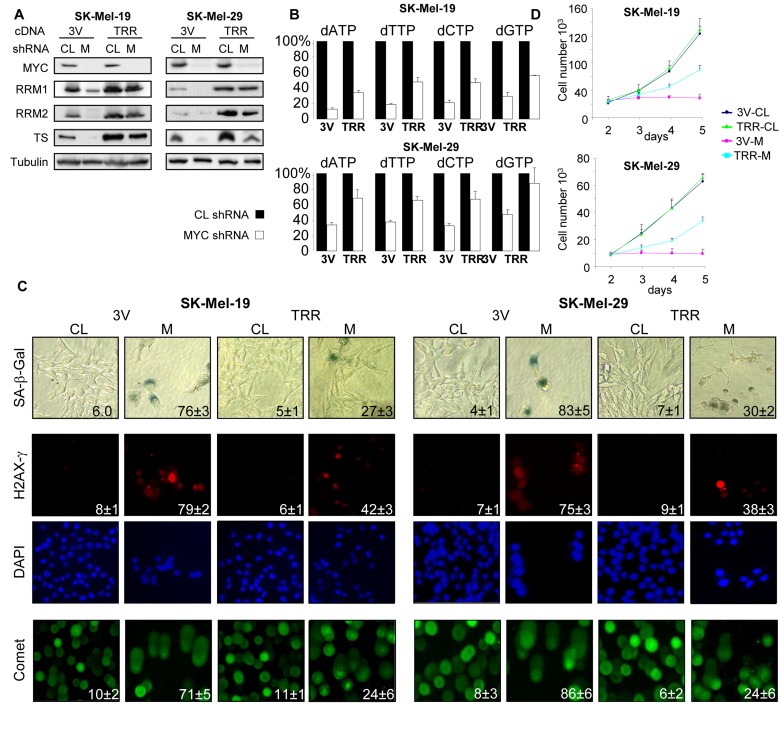
Ectopic expression of TS, RRM1 and RRM2 suppress senescence in MYC-depleted melanoma cells (**A**) Cells were infected three times with control vectors (3V) or with vectors expressing TS, RRM1 and RRM2 cDNAs (TRR). Forty-eight hours later, cells were infected again with control shRNA (CL) or MYC shRNA (M). 5 days after the second infection, cells were collected and total protein lysates were probed in western blotting with the antibodies indicated on the left. (**B**) Cells were infected as described above. Five days after infection dNTPs were extracted and quantified. dNTP amounts were normalized by the amounts detected in 3V-CL cells or TRR-CL cells. (**C**) Cells were infected as described in (**A**). Five days after infection, cells were processed to detect SA-β-Gal activity, or stained with H2AX-γ-specific antibodies and DAPI or were used in the comet assay. Percentage of positive cells is indicated. (**D**) Cells that were infected as was described in (**A**) were plated in 12-well plates and counted every day for 3 days. Numbers below the graph correspond to the days post-infection.

Based on the above data, we hypothesized that senescence in MYC-depleted cells was induced by low expression of TS, RRM1 and RRM2 that led to decreased amounts of intracellular dNTPs and subsequently to DNA damage. To test this hypothesis, we concurrently infected SK-Mel-19 and SK-Mel-29 cells with lentiviral vectors containing cDNAs for TS, RRM1 and RRM2 (TRR) or with three empty vectors (3V). 48 hours post-infection, cells were super-infected with control or C-MYC shRNA-expressing viruses. As shown in Figure 2AB, levels of TS, RRM1 and RRM2 proteins as well as the amounts of dNTP pools were significantly higher in C-MYC-depleted TRR-cells than in 3V cells. Similarly, other senescence-associated phenotypes, including DNA damage, activity of SA-β-Gal and proliferation arrest were substantially less pronounced in TRR-MYC than in 3V-MYC populations of cells (Figure 2CD). Therefore, co-expression of TS, RRM1 and RRM2 in melanoma cells partially overcomes senescence caused by C-MYC depletion.

### Supplementation with deoxyribonucleosides suppresses senescence phenotypes in MYC-depleted melanoma cells

**Figure 3:**
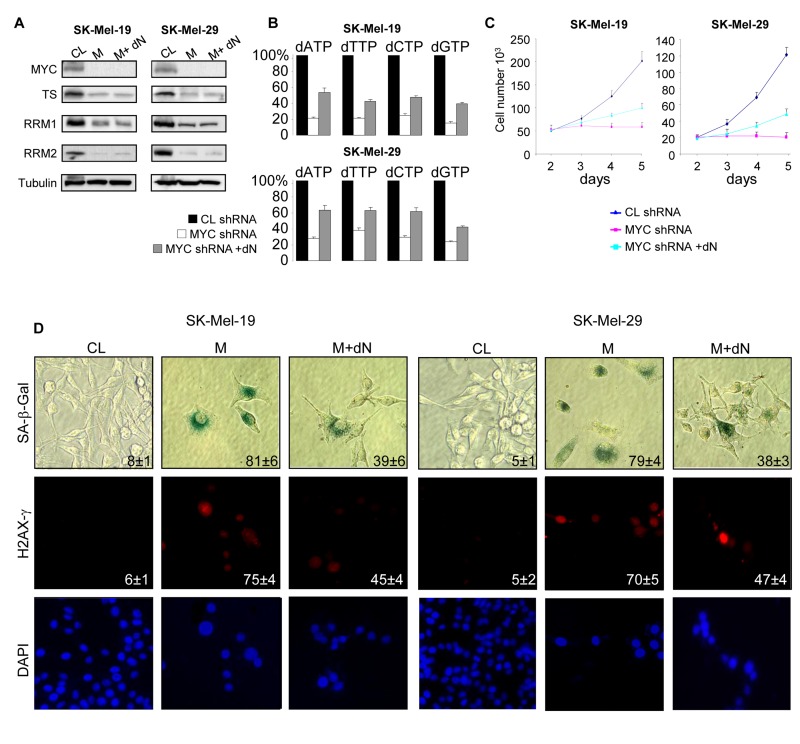
Addition of deoxyribonucleosides reduces senescence phenotypes in MYC-depleted melanoma cells (**A**) Cells were infected with control shRNA (CL) or MYC-shRNA (M) following by incubation with control media or media containing a mixture of 4 deoxyribonucleosides (100 μM each) (M+dN). Five days after infection, cells were collected and total protein lysates were probed in western blotting with the antibodies shown on the left. (**B**) Cells were infected and treated as described in (**A**). dNTPs were extracted 3 days after infection and quantified. All dNTP amounts were normalized by the amounts detected in CL cells. (**C**) Cells were infected and treated as described in (**A**). The second day after infection, cells were plated in 12-well plate in and counted every day for 5 days. (**D**) Cells were infected as was described in (**A**). Five days after infection, cells were processed to detect SA-β-Gal activity, or stained with H2AX-γ-specific antibodies and DAPI. The percentage of positive cells is indicated. Numbers below the graph correspond to the days post-infection.

To evaluate the role of enhanced dNTP pools in MYC-dependent suppression of senescence, MYC-depleted SK-Mel-19 and SK-Mel-29 cells were incubated in growth media supplemented with deoxyadenosine, thymidine, deoxycytidine and deoxyguanosine (100 μM of each). The deoxyribonucleosides were added next day after infection with MYC shRNA, and culture media with and without deoxyribonucleosides were refreshed every 2 days. Treatment with exogenous deoxyribonucleosides did not alter levels of C-MYC, TS, RRM1 or RRM2 (Figure 3A), however it increased the amounts of intracellular dNTPs (Figure 3B) and suppressed senescence phenotypes including DNA damage, activity of SA-β-Gal and inhibition of proliferation (Figure 3CD).

Taken together, our data indicate that depletion of dNTPs is one of the major causes of DNA damage and senescence in MYC-depleted melanoma cells and that maintaining high TS, RRM1 and RRM2 levels is important for C-MYC-dependent suppression of senescence programs in these cells.

## DISCUSSION

Recently, we and others have demonstrated that acute depletion of C-MYC in melanoma cells resulted in proliferation arrest that strikingly resembled oncogene-induced senescence in normal cells [6]. Moreover, like in normal cells, senescence phenotypes in MYC-depleted melanoma cells depended on constitutively active BRAF or NRAS oncoproteins [6], but not on p53 or p16^INK4A^ status [13]. And although a crosstalk between Cdk2 and C-MYC was implicated in C-MYC induction and maintenance of senescence [14], C-MYC target genes responsible for continuous suppression of senescence in melanoma cells were not identified [6, 10]. Our present data demonstrate that at least one of the major causes of senescence in MYC-depleted melanoma cells is the down-regulation of TS- and RR-dependent dNTP pools.

TS and RRM2 have been previously identified as E2F1-responsive genes [15]. Recently, we and others have demonstrated that TS, RRM1 and RRM2 are also direct C-MYC-targets [10, 16]. E2F1 levels were not altered by MYC-depletion in melanoma cells [10] indicating that under-expression of TS and RR and depletion of dNTP pools in senescent melanoma cells is likely to be a direct consequence of C-MYC suppression.

Senescence-like phenotypes including proliferation arrest were induced in melanoma cells by the inhibition of oncogenic chromatin-remodeling factor DEK [17] or microphthalmia-associated transcription factor MITF, a master regulator of melanocyte homeostasis and melanomagenesis [18-20]. It has been reported that MITF-depleted senescent cells activated the DNA damage response [18], whereas silencing of DEK increased sensitivity of melanoma cells to the DNA damaging agents [17]. Thus, it will be of interest to test whether DNA damage *per se* or susceptibility to DNA damage caused by inhibition of DEK or MITF in melanoma cells are mediated by the depletion of dNTP pools, and whether upregulation of TS and RR suppresses these phenotypes.

Induction of senescence in tumors is currently being considered as an alternative outcome of conventional chemotherapeutic approaches [4, 5]. The molecular mechanisms underlying emergence or re-activation of senescence programs in transformed cells still remain elusive. Our data have uncovered novel mechanisms for the induction of DNA damage and senescence in melanoma cells that may be applicable to senescence phenotypes in non-melanoma tumor cells.

## METHODS

### Cell lines and populations

Melanoma cell lines were obtained from Memorial Sloan Kettering Cancer Center. Cells were cultured in Dulbecco's modified Eagle's essential minimal medium supplemented with fetal calf serum (10%), 2 mM glutamine, and a mixture of penicillin and streptomycin.

### Lentiviral constructs and infection

Lentiviral infection and vectors containing control shRNA, C-MYC shRNA, RRM2 shRNA, and cDNAs for TS, RRM1 and RRM2 were described previously [6, 10, 12].

### Assays for cell senescence

Cells were fixed and stained using Senescence detection kit (Biovision) according to the manufacturer's recommendations.

### Immunoblotting, immunofluorescence and comet assay

The following antibodies were used in western blotting: C-MYC, RRM1, RRM2 from Santa Cruz Biotechnology (sc-42, sc-11733, sc-10844, respectively) and TS (ab7398-1) from Abcam. Membranes were developed using alkaline phosphatase-conjugated secondary antibodies and the Alpha-Innotech FluorChem HD2^®^ imaging system. For immunofluorescence, formaldehyde-fixed cells were stained with H2AX-γ-specific antibodies (05-636, Millipore) in accordance with the manufacturer's recommendations. Alkaline comet assay was performed using Trevigen CometAssay® HT kit (4252-040-K) according to the manufacturer's recommendations.

### Quantification of deoxyribonucleotide triphosphates

dNTPs were extracted and assayed by the DNA polymerase-based method as described previously [10].
